# Supplementary material for: Association between shift work in early pregnancy, snacking, and inappropriate weight gain during pregnancy: The Japan Environment and Children’s Study
Source: PLoS One. 2023 Oct 12;18(10):e0291579. doi: 10.1371/journal.pone.0291579 (PMC10569563; doi:10.1371/journal.pone.0291579)
Supplement: Attachment — Submitted filename: General comment.docx [file pone.0291579.s002.docx]

**General comment**

The work by Dr. Funakai and colleagues addresses an important issue: the association between shift work and inappropriate weight gain during pregnancy. Specifically, longitudinal data are used to examine the association between shift work in early pregnancy, snacking, and inappropriate weight gain during pregnancy. The authors conclude that pregnant women who work one or more shifts per month during early pregnancy consume fast food, potato chips, and other snack foods more frequently than pregnant women who work only day shifts. Of note, changing the definition of shift work and the definition of the cut-off points for habitual consumption of fast food, snacks, and late-night snacks did not significantly change the results.

**Major comments**

1. Energy intake is one of the most important factors in weight gain. However, this study appears to have conducted a food frequency survey but did not take energy intake into account when analyzing the data. Energy intake should be taken into account.
2. The authors note that there is no clear dose-response relationship between night work and metabolic diseases such as diabetes. Conversely, a previous study reported a dose-response relationship between the number of night shifts per month and the risk of diabetes (Céline Vetter et al., Diabetes Care, 41(4):762-769, 2018). In the present study, no clear association was found between night shifts and their frequency during pregnancy and weight gain. For the reader's understanding, this finding should be discussed in more detail. Please provide a citation.
3. The authors state in the Introduction that there is no evidence that shift work causes inappropriate weight gain or unhealthy snacking during pregnancy (lines 62-64). However, the authors discuss a previous study in the Discussion section of the manuscript (lines 282-284). The manuscript should be revised in the Introduction due to this inconsistency.

**Minor comments**

1. The tables are generally difficult to read and should be corrected. In particular, the tables need to be modified so that they fit into the manuscript.
2. In Table 2, there is a missing number in “Pregnant women who gained too little weight during pregnancy”.
3. Is the percentage of "Pregnant women whose weight gain was in the inappropriate range during pregnancy" the combined value of the following results? There are some incorrect values (Table 2).
4. Should not the physical activity levels be included in the results along with the other covariates?
5. Please provide a citation (lines 282-284).
