# Supplementary material for: Association between shift work in early pregnancy, snacking, and inappropriate weight gain during pregnancy: The Japan Environment and Children’s Study
Source: PLoS One. 2023 Oct 12;18(10):e0291579. doi: 10.1371/journal.pone.0291579 (PMC10569563; doi:10.1371/journal.pone.0291579)
Supplement: Attachment — Submitted filename: Response to Reviewers.docx [file pone.0291579.s003.docx]

Dear Dr. Yamada,

Thank you for your ongoing consideration of our manuscript for publication in *PLOS ONE*. We appreciate the time you and the reviewers spent and believe the revised manuscript is improved. Below, we have addressed your and the reviewers’ comments. The journal requirements and reviewer’s comments are in bold and numbered.

The tables and the figures have been amended, and we hope they are now clearer. I have considered all the comments and suggestions made by reviewers of this paper, and I have incorporated most of them in the final version of this paper.

Please find the revised manuscript suitable for publication in *PLOS ONE*.

We would like to thank you the reviewers once more for sparing the time to write so many detailed and valuable comments.

With kind regards,

Satomi Funaki-Ishizu, MD

Division of Health Administration and Promotion, Department of Social Medicine, Faculty of Medicine, Tottori University (on behalf of all the authors)

**Journal Requirements:**

**1. Please ensure that your manuscript meets PLOS ONE's style requirements, including those for file naming.**

[Response]

As suggested, we have changed the manuscript format and file naming the title to PLOS ONE's style requirements.

**2. Thank you for stating the following financial disclosure:**

**“no”**

**At this time, please address the following queries:**

**a) Please clarify the sources of funding (financial or material support) for your study. List the grants or organizations that supported your study, including funding received from your institution.**

[Response]

We appreciate your comments and the opportunity to clarify this critical point. This study was funded by the Ministry of Environment, Japan.

**b) State what role the funders took in the study. If the funders had no role in your study, please state: “The funders had no role in study design, data collection and analysis, decision to publish, or preparation of the manuscript.”**

[Response]

We thank the editor for the positive comment. The funder had no role in the design and conduct of the study; collection, management, analysis, and interpretation of the data; preparation, review, or approval of the manuscript; and decision to submit the manuscript for publication.

**c) If any authors received a salary from any of your funders, please state which authors and which funders.**

[Response]

No. The authors received no salary from funders.

**d) If you did not receive any funding for this study, please state: “The authors received no specific funding for this work.”**

[Response]

The authors received no specific funding for this work.

**Please include your amended statements within your cover letter; we will change the online submission form on your behalf.**

**3. Thank you for stating the following in your Competing Interests section:**

**“no”**

**Please complete your Competing Interests on the online submission form to state any Competing Interests. If you have no competing interests, please state "The authors have declared that no competing interests exist.", as detailed online in our guide for authors at http://journals.plos.org/plosone/s/submit-now**

**This information should be included in your cover letter; we will change the online submission form on your behalf.**

[Response]

The authors have declared that no competing interests exist.

**4. Thank you for stating the following in the Acknowledgments Section of your manuscript:**

**“This study was funded by the Ministry of the Environment, Japan. The findings and conclusions of this article are solely the responsibility of the authors and do not represent the official views of the above government. We thank Audrey Holmes, MA, and Benjamin Knight, MSc., from Edanz (https://jp.edanz.com/ac) for editing a draft of this manuscript.”**

**We note that you have provided additional information within the Acknowledgements Section that is not currently declared in your Funding Statement. Please note that funding information should not appear in the Acknowledgments section or other areas of your manuscript. We will only publish funding information present in the Funding Statement section of the online submission form.**

**Please remove any funding-related text from the manuscript and let us know how you would like to update your Funding Statement. Currently, your Funding Statement reads as follows:**

**“NO”**

**Please include your amended statements within your cover letter; we will change the online submission form on your behalf.**

[Response]

We thank the editor for the careful review. As suggested, we have removed the Funding Statement section and updated our Funding Statement.

**5. Your ethics statement should only appear in the Methods section of your manuscript. If your ethics statement is written in any section besides the Methods, please delete it from any other section.**

[Response]

We appreciate the editor for the careful review. As suggested, we have removed the ethics statement from other sections besides the Methods.

**5. Review Comments to the Author**

**Please use the space provided to explain your answers to the questions above. You may also include additional comments for the author, including concerns about dual publication, research ethics, or publication ethics. (Please upload your review as an attachment if it exceeds 20,000 characters)**

**Reviewer #1:**

**This paper thoroughly examined the relationship between night shift work in early pregnancy and weight gain as well as snacking behavior during pregnancy, using large-scale data sets. The manuscript is well-organized and clearly presents the results, which provide valuable information for understanding the dietary challenges faced by pregnant women working night shifts. I think the sensitivity analysis has been carefully performed and the limitations have been well considered. However, one aspect that requires clarification is the analysis of the association between night shift work in early pregnancy and pre-pregnancy snacking behavior. The significance and interpretation of this relationship were somewhat unclear.**

**Major Concerns**

1. **The authors state their objective as "investigating the effects of night shifts on snacking behaviors during pregnancy," indicating their interest in exploring causal relationships. However, the analysis examines the relationship between night shifts in early pregnancy and the frequency of fast food consumption in the 20s prior to pregnancy, as well as the frequency of snack consumption such as potato chips in the year before pregnancy. Given the strict temporal ordering, this analysis appears to be inconsistent with the objective of investigating the 'effects' on snacking behaviors during pregnancy, as pre-pregnancy eating habits precede night work in early pregnancy. If the authors cannot provide justification for this analysis, it would be advisable to consider excluding the results pertaining to snacking behaviors before pregnancy from Table 3. Alternatively, the frequency of fast food and snack intake before pregnancy could be positioned as a baseline and used as an adjustment factor when examining the effects of night shifts in early pregnancy on snacking behavior in mid-to-late pregnancy.**

[Response]

We thank the reviewer for these excellent comments. We agree with the reviewer’s observation that "pre-pregnancy eating habits precede night work in early pregnancy" and have removed snacking behaviors before pregnancy from Table 3.

**Minor**

2. **Abstract & L27: 'evening meal'**

**Is the term 'evening meal' synonymous with 'midnight snack'? Please ensure consistent terminology.**

[Response]

We have carefully reviewed the manuscript and corrected typos.

3. **L156: 'skipped breakfast less than three times a week were defined as habitually missing breakfast.'**

**I believe 'less than' should be replaced with 'more than' or 'at least.'**

[Response]

As suggested, we have changed the expression to "We defined those who said they skipped breakfast more than thrice a week as habitually missing it."

**L163:**

4. **In this paper, the emphasis is solely on potato chips for snacking behavior. It was unclear whether the questionnaire included other types of snacks apart from potato chips. Providing more detailed explanations in the methods section would help clarify this aspect.**

[Response]

We thank the reviewer for the positive comment. As the reviewer suggested, we carefully checked the questionnaire. The questionnaire 'Snack foods' included other snack foods besides potato chips. We added information about the definition of snack foods in the section "Frequency of consuming snack foods" (page 11, para 3.). We also discussed this issue in the Limitation section(page 31, para 1.)

5. **Table 2:**

**Some variable names begin with '-ing,' while others begin with 'Frequency.' It would be preferable to standardize the wording throughout the table, unless there is a specific intention behind this variation.**

[Response]

We have carefully reviewed and standardized the variable wordings.

**Reviewer #2: General comment**

**The work by Dr. Funaki and colleagues addresses an important issue: the association between shift work and inappropriate weight gain during pregnancy. Specifically, longitudinal data are used to examine the association between shift work in early pregnancy, snacking, and inappropriate weight gain during pregnancy. The authors conclude that pregnant women who work one or more shifts per month during early pregnancy consume fast food, potato chips, and other snack foods more frequently than pregnant women who work only day shifts. Of note, changing the definition of shift work and the definition of the cut-off points for habitual consumption of fast food, snacks, and late-night snacks did not significantly change the results.**

**Major comments**

**1. Energy intake is one of the most important factors in weight gain. However, this study appears to have conducted a food frequency survey but did not take energy intake into account when analyzing the data. Energy intake should be taken into account.**

[Response]

We thank the reviewer for these excellent comments. As requested, we have added this information to the result section (page 20, para 2).

**2. The authors note that there is no clear dose-response relationship between night work and metabolic diseases such as diabetes. Conversely, a previous study reported a dose-response relationship between the number of night shifts per month and the risk of diabetes (Céline Vetter et al., Diabetes Care, 41(4):762-769, 2018). In the present study, no clear association was found between night shifts and their frequency during pregnancy and weight gain. For the reader's understanding, this finding should be discussed in more detail. Please provide a citation.**

[Response]

We appreciate the reviewer's careful review. As requested, we have added this citation to the Introduction section. (page 3, para 2).

**3. The authors state in the Introduction that there is no evidence that shift work causes inappropriate weight gain or unhealthy snacking during pregnancy (lines 62-64). However, the authors discuss a previous study in the Discussion section of the manuscript (lines 282-284). The manuscript should be revised in the Introduction due to this inconsistency.**

[Response]

We appreciate the opportunity to clarify this point. There is much previous research on shift work and dietary habits, obesity, and non-communicable diseases such as hypertension and diabetes. However, most studies did not focus on gestational women as primary participants. In addition to the discussion section, we clarified that our study found no significant association between night shift work and inappropriate weight gain in pregnancy and discussed this issue (page 27, para 2).

**Minor comments**

**1. The tables are generally difficult to read and should be corrected. In particular, the tables need to be modified so that they fit into the manuscript.**

[Response]

We have organized and modified the new tables to fit the manuscript.

**2. In Table 2, there is a missing number in “Pregnant women who gained too little weight during pregnancy”.**

[Response]

We added the number of “Pregnant women who gained too little weight during pregnancy” to Table 1.

**3. Is the percentage of "Pregnant women whose weight gain was in the inappropriate range during pregnancy" the combined value of the following results? There are some incorrect values (Table 2).**

[Response]

We thank the reviewer for the careful review and corrected the value.

**4. Should not the physical activity levels be included in the results along with the other covariates?**

[Response]

We thank the reviewer for the positive comment. We added the results, physical activity levels, with other covariates in Table 4.

**5. Please provide a citation (lines 282-284).**

[Response]

Please see reviewer #2’s major comment No.3 and our comment.
